# Role of Extracellular Actin and Its Clearance System in Immune Mechanisms of Disease Development

**DOI:** 10.3390/ijms272110049

**Published:** 2026-11-09

**Authors:** Nikolay P. Sudakov, Ekaterina V. Moskaleva, Lyubov V. Rychkova, Sergey I. Kolesnikov, Natalya V. Semenova, Lyubov I. Kolesnikova

**Affiliations:** Scientific Centre for Family Health and Human Reproduction Problems, 664003 Irkutsk, Russia; mkatena@mail.ru (E.V.M.); iphr@sbamsr.irk.ru (L.V.R.); sikolesnikov2012@gmail.com (S.I.K.); natkor_84@mail.ru (N.V.S.); kolesnikova20121@mail.ru (L.I.K.)

**Keywords:** actin, gelsolin, Gc-globulin, thymosin β4, profilin-1, anti-actin autoantibodies

## Abstract

This review focuses on the mechanisms by which extracellular actin and its clearance system components contribute to the development of pathological processes. Actin can be released from cells under conditions of stress, injury, or death. In the extracellular environment, actin exerts diverse effects on the immune and hemostatic systems. To remove excess extracellular actin, the body employs an elimination system that includes plasma gelsolin, Gc-globulin, thymosin β4, and profilin-1. The specific effect depends on the concentration of F-actin. At low, physiological concentrations—effectively managed by the clearance system—actin participates in the cross-presentation of intracellular antigens, the local stabilization of thrombi, the formation of extracellular traps, and the activity of antibacterial peptides. At higher, uncompensated concentrations, actin leads to the excessive stabilization of extracellular traps and thrombi against degradation, resulting in hyperinflammation, immunothrombosis, and increased susceptibility to infections. Furthermore, in predisposed individuals, this can lead to the development of autoantibodies against actin, contributing to various autoimmune processes. A complete loss of control over extracellular actin levels results in a sharp spike in actin concentration, causing vascular damage and obstruction, thrombosis, and, ultimately, death. Investigating the interactions between extracellular actin and the proteins responsible for its removal will help improve the diagnosis and treatment of many socially significant diseases.

## 1. Introduction

The actin protein (G-actin, 42 kDa) is the main structural component of the actin microfilament system, which is part of the cytoskeleton. This system is represented by filaments of polymerized actin (F-actin) in complex with actin-binding proteins. Depending on the function, they stabilize, depolymerize, combine microfilaments into various structures, or move along them [1]. The actin cytoskeleton is highly dynamic [2,3], involved in the motility of cells and their organelles [4,5,6], contacts of cells with each other [7] and with components of the extracellular environment [8,9]. Reorganizations of the actin skeleton underlie changes in the functional activity and differentiation of cells [10]. The role of this system in division [6] и and programmed cell death [11,12,13,14,15] has also been shown. Under stressful and damaging influences on cells, F-actin is capable of adaptive [16,17] and degenerative restructuring [18,19]. In this case, oxidative stress, which is a non-specific component of many pathological processes [20,21,22,23,24], actively interacts with the microfilament system: the cysteine residues C285 and C374 in the actin molecule participate in antioxidant protection, and oxidized actin in the cell forms specific granular structures (oxidation-induced actin bodies) [25]. In general, the actin cytoskeleton is involved in the mechanisms of development of various diseases: inflammatory [26,27], oncological processes (cell transformation, invasion and metastasis) [28,29,30], fibrotic changes in tissues [31,32], and regeneration processes [33,34,35]. The relationship of the actin cytoskeleton with pathogenic microorganisms is interesting. For intracellular parasites (bacteria, viruses), the ability to use the system of actin microfilaments of the cell to ensure their own vital activity has been shown [36]. On the other hand, an important role of this system in the processes of recognition of bacteria and mechanisms of protection against them has been established [37].

Like other intracellular proteins [38,39], actin can be released from cells when they are damaged or stressed and can be found in various extracellular environments of the body [40,41,42,43,44,45]. As a marker of damaged cells, in physiologically acceptable quantities, F-actin induces normal responses to tissue damage: it stimulates inflammation, enhances the immune response to foreign antigens [46]. Excessive amounts of F-actin in the extracellular environment and insufficiency of its clearance system lead to the development of its negative effects: endothelial damage, thrombosis, induction of inflammation, immune response and autoimmune reactions [47]. These, primarily immunological or immune-mediated, processes are pathogenetic links in the development of many diseases in humans and their complications, causing limitation or loss of ability to work, disability and death. In this regard, this review is devoted to a detailed analysis of the interactions of extracellular actin and the proteins that carry out its clearance, with various components of the immune system, which is important for understanding the mechanisms of participation of damage, stress and cell death in the development of socially significant diseases and their complications.

## 2. F-Actin in the Extracellular Environment and Its Clearance Mechanisms

It has been established that actin is capable of entering the extracellular space [45] and the systemic circulation: blood, lymph, cerebrospinal fluid [48]. Actin has also been detected in urine [49] and bronchial alveolar fluid [50]. Production of both polymerized actin and its monomers from dead, damaged or stressed cells is possible [47]. The simplest mechanism for the appearance of actin outside the cell is the release of this protein from damaged cells. The production of actin by stressed cells that retain their structural integrity can occur through the budding of microvesicles and the shedding of cellular processes [51]. The possibility of transmembrane actin transport is a puzzling phenomenon, as actin lacks a signal peptide for membrane translocation or a transmembrane domain (unlike gelsolin) [52]. Nevertheless, there are mechanisms for the import and export of actin across the nuclear envelope, where actin is transported in a complex with cofilin, which contains a transport sequence [53]. Although transport across the nuclear envelope—mediated by nuclear pores—is difficult to compare to transport across the plasma membrane, it is plausible that mechanisms exist for the transport of actin in complex with other proteins that act as carriers. Due to the presence of ions and ATP in the extracellular environment, G-actin is capable of immediate self-assembly into microfilaments [54]. For example, the formation of large F-actin aggregates exceeding cell size has been demonstrated in the intervertebral disc matrix during degenerative processes [45,55], as well as following epidermal injury [56]. The appearance of F-actin in the blood has been established in patients with myocardial infarction [57], angina pectoris [42], severe trauma [58], liver necrosis and septic shock [59,60,61], and necrotizing enterocolitis [62]. Moreover, extracellular actin microfilaments are not only evidence of cell damage in the body, but also have a pronounced negative effect. The question of how actin enters the systemic circulation is highly intriguing. To date, there are no studies that clearly delineate the mechanisms underlying this process. We hypothesize that actin might enter these extracellular environments either through the destruction of endothelial cells or via active production of actin by these cells. It is also plausible that the protein crosses the endothelial barrier from surrounding tissues—whether through damaged blood vessel areas or through undamaged vessel areas—via endothelial fenestrae, transcytosis, or by the opening of tight junctions [47]. It has been revealed that freely circulating F-actin can cause a lethal outcome due to changes in blood viscosity and obstruction of small vessels, activation of thrombus formation [63] and impairment of the fibrinolysis process [58,64,65], damage to the endothelium [48,54] and the formation of microaneurysms [66]. Experimental intravenous infusions of G-actin in rats have objectively shown these harmful effects of extracellular actin at high levels. In this work, the injection of 14 mg of G-actin led to sudden cardiopulmonary arrest and right heart dilatation with fatal outcome [54]. Pathologic examination of these rats has shown all effects discussed above (vascular damage, obstruction, and thrombosis). The pronounced negative effects of actin microfilaments necessitate their removal; to this end, an F-actin clearance system exists in the extracellular environment (Figure 1; Supplementary Video S1). To date, two of its main specialized components have been identified: gelsolin [67] and Gc-globulin (Gc-globulin: group-specific component, also known as vitamin D-binding protein) [68,69]. The function of gelsolin (82–84 kDa depending on the isoform) is the depolymerization of F-actin to G-actin. This process is realized through the binding, cutting and capping of actin microfilaments. Capping of F-actin occurs at its plus end, to which actin monomers are predominantly attached in a constant process of self-assembly. Binding of the plus end of the microfilament by gelsolin blocks the attachment of G-actin to it, while actin monomers from the free minus end continue to dissociate. This leads to complete depolymerization of F-actin [70]. Gelsolin is localized mainly inside cells—in the cytosol, where it, in concert with other actin-binding proteins, ensures the rearrangement of the actin cytoskeleton [71,72,73]. There is an extracellular secreted form of this protein—plasma-type gelsolin (pGSN), designed for the clearance of F-actin [74,75]. The main site of production of plasma gelsolin is skeletal, cardiac and smooth muscle tissue [76,77,78]. Gc-globulin (58 kDa) captures actin monomers and promotes their removal from the systemic circulation through liver cells [59,79,80,81]. This protein belongs to the albumin family [82]. The site of synthesis of Gc-globulin is the liver [83]. In addition to G-actin clearance, this protein is the main transporter of vitamin D and its metabolites in the blood [84]. The independent arrangement of the domains for interaction with actin and vitamin D in the Gc-globulin molecule allows for the simultaneous attachment of both of these ligands [85]. Thymosin β 4 (4.96 kDa) [86,87,88,89] and profilin-1 (12–16 kDa) [90] can also be attributed to the extracellular actin clearance system. In the body, there are no forms of these proteins specialized for extracellular localization. In the cell, these molecules bind and sequester G-actin to reduce its free pool used for polymerization [91]. Outside the cell, they can also form complexes with G-actin. The predominant source of these proteins in the extracellular environment is activated platelets [90,92,93] and, to a lesser extent, damaged cells [94]. Other sources of active production of these proteins are also possible. Thus, it has been shown that in response to glucocorticoids, monocytes produce thymosin β4 sulfoxide [95], which inhibits actin polymerization 20 times more weakly [96]. The presence of profilin 1 in the composition of exosomes released from the surface of various cells has been established [97]. In comparison with Gc-globulin, the ability to bind G-actin in profilin 1 [82] and thymosin β4 [98] is significantly less. This is a consequence of the specialization of these proteins in the temporary sequestration of free G-actin in the cytosol, which predetermines their ability to quickly release bound actin monomers to form new microfilaments in the cell [99]. Moreover, for profilin 1, the fact of transfer of actin monomers bound to it to Gc-globulin has been established [82]. This, taking into account the equally low binding capacity of actin monomers, may also be characteristic of thymosin β4. The affinity of Gc-globulin for G-actin is so high that it is maintained when the pH and ionic composition of the microenvironment changes, and also when a three-component complex with DNase I (DNase I—Gc-globulin—G-actin) is formed [82].

In general, actin release into the extracellular space is a common event in the body, accompanying stress, injury, and cell death. In this location, actin is predominantly present in a polymerized form, as it has the ability to self-assemble. High concentrations of this form of actin cause vascular damage and obstruction, as well as thrombosis, which can be fatal. Therefore, actin release into the extracellular space is compensated for by the body’s coordinated recycling system.

## 3. Changes in the Level of Components of the Actin Clearance System in Pathological Processes

F-actin recorded in various diseases may be a consequence of the insufficiency of its clearance system (Table 1). A decrease in the gelsolin level is associated with an increased likelihood of mortality and the development of complications in patients in intensive care units [100], hemodialysis [101,102,103], after stroke [104], severe burns and injuries [43] with sepsis [60], community-acquired pneumonia (CAP) [105], COVID-19 [106,107,108]. It has been established that after hematopoietic stem cell transplantation, a decrease in this indicator is a predictor of the development of idiopathic pneumonia [109]. A decrease in the concentration of gelsolin in the blood has been detected in tick-borne encephalitis and Lyme borreliosis [110], and malaria caused by *Plasmodium falciparum* [111]. Hypogelsolinemia is also observed in inflammatory processes: multiple sclerosis [112], rheumatoid [113] and psoriatic arthritis [114], atopic dermatitis [115]. Autoimmune processes characterized by a decrease in the concentration of gelsolin have been identified: IgA nephropathy [116], systemic lupus erythematosus [117]. A decrease in the level of gelsolin in the blood has been shown in allergic rhinitis [118]. The level of this protein in plasma decreases in conditions of preeclampsia [119], in newborns with bronchopulmonary dysplasia [120], in chronic hypoxia [121], and in hypoxic-ischemic encephalopathy [122]. The reason for the decrease in the concentration of gelsolin in the blood may be an increase in extracellular actin [43] and modulation of the gelsolin level by various inflammatory mediators [104]. Elevated levels of gelsolin in the blood have been recorded in patients with HIV-1, and its inverse correlation with the number of CD4(+) cells is characteristic [123]. The concentration of this protein in the plasma of patients receiving chronic hemodialysis is also increased [124].

A decrease in the concentration of Gc-globulin is detected in the blood of patients with impaired liver function, since this organ is the main site of its production [125]. Thus, a decrease in the level of this protein has been shown in fulminant liver necrosis [126], acute liver failure [127], and non-alcoholic fatty liver disease [128]. Also, the concentration of Gc-globulin in the blood decreases with severe injuries [129] and septic shock [130]. Its decrease has been shown in multiple sclerosis [84,131] and systemic lupus erythematosus [132]. The level of this protein in the blood increases one week after severe injuries [133]. High levels of Gc-globulin are characteristic of patients in chronic hemodialysis units, and a decrease in it is associated with an increased likelihood of a fatal outcome [134].

Thymosin β4 levels have been shown to decrease in sepsis [87], but this has no predictive value [135]. Low values of this blood parameter have been identified in non-alcoholic fatty liver disease [136]; an inverse correlation exists with the histological activity of the process and the degree of oxidative stress [137]. Decreased thymosin β4 concentrations are associated with increased mortality in patients with chronic hepatitis B virus [138]. Thymosin β4 has been established to be produced by activated platelets, and its amount increases in areas of tissue damage [139]. Its level has been shown to increase in the blood during myocardial infarction [140] and is one of the markers of its development [141]. On the other hand, in patients with myocardial infarction who received experimental intramyocardial injections of bone marrow stem cells characterized by an increase in the concentration of thymosin β4 in the blood, an improvement in the NHYA symptom scale was observed after 6 months [142]. Apparently, such a delayed positive effect of an increased level of thymosin β4 is associated with its antifibrotic and angiogenic activity [143,144]. A high concentration of thymosin β4 has been detected in osteoarthritis of the knee joint, and a direct correlation of this indicator with the severity of the process has been shown [145].

Elevated serum profilin-1 levels are associated with endothelial dysfunction, cardiovascular events, and high mortality in patients with chronic kidney disease [146]. Blood profilin-1 concentrations are also increased in pre-eclampsia, associated with vascular dysfunction [147], and in patients on chronic hemodialysis, directly correlating with vascular intima-media thickness [148].

Thus, blood levels of extracellular actin clearance system components can either decrease or increase. A decrease may result from system depletion due to intense complexation with actin or from impaired production. As mentioned above, a decrease in the concentration of these proteins will contribute to an increase in F-actin levels and its negative effects. An increase in actin clearance system components may be a compensatory mechanism in response to actin release from cells or may be due to a response to tissue damage.

**Table 1:** Components of the extracellular actin clearance system and their level changes in diseases.

Protein	Source	Function	Affinity (K_d_) * for G-Actin	
**Plasma gelsolin** (84 kDa)	Skeletal, cardiac and smooth muscle tissue [76,77,78]	Depolymerization of F-actin to G-actin	2.0 nM [149]	***decrease in the gelsolin level***
				Severe burns and injuries [43]
				Stroke [104]
				Patients in intensive care units [100]
				Chronic hemodialysis mortality [101,102,103]
				Severe sepsis [60]
				Rheumatoid arthritis [113]
				Psoriatic arthritis [114]
				Atopic dermatitis [115]
				Multiple sclerosis [112]
				Community-acquired pneumonia [105]
				Idiopathic pneumonia [109]
				COVID-19 [106,107,108]
				Malaria caused by *Plasmodium falciparum* [111]
				Tick-borne encephalitis [110]
				Lyme borreliosis [110]
				IgA nephropathy [116]
				Systemic lupus erythematosus [117]
				Allergic rhinitis [118]
				Preeclampsia [119]
				Newborns with bronchopulmonary dysplasia [120]
				Newborns with chronic hypoxia [121]
				Newborns with hypoxic-ischemic encephalopathy [122]
				***increase in the gelsolin level***
				Patients with HIV-1 (with inverse correlation with the number of CD4(+) cells) [123]
				Patients on chronic hemodialysis [124]
**Gc-globulin** (58 kDa)	Liver [83]	Captures G-actin and promotes its removal from the systemic circulation through liver cells [59,79,80,81]	1.0 nM [150]	***decrease in the Gc-globulin level***
				Fulminant liver necrosis [126]
				Acute liver failure [127]
				Non-alcoholic fatty liver disease [128]
				Severe injuries [129]
				Septic shock [130]
				Multiple sclerosis [84,131]
				systemic lupus erythematosus [132]
				Patients in chronic hemodialysis units with an increased likelihood of a fatal outcome [134]
				***increase in the Gc-globulin level***
				Patients in chronic hemodialysis units [134]
				Patients one week after severe injuries [133]
**thymosin β4** (4.96 kDa)	platelets [90,92,93], damaged cells [94]	sequester G-actin to reduce its free pool used for polymerization and transfer it to Gc-globulin	0.5 to 2.0 μM [151]	***decrease in the thymosin β4 level***
				Sepsis [87]
				Non-alcoholic fatty liver disease [136]
				Mortality in chronic hepatitis B virus [138]
				***increase in the thymosin β4 level***
				Myocardial infarction [140]
				osteoarthritis of the knee joint [145]
**profilin-1** (12–16 kDa)	platelets [90,92,93], damaged cells [94]	sequester G-actin to reduce its free pool used for polymerization and transfer it to Gc-globulin	0.1 to 1.0 0 μM [152]	***decrease in the profilin-1 level***
				There is no information about this.
				***increase in the profilin-1 level***
				Mortality in chronic kidney disease [146]
				Preeclampsia [147]
				Patients on chronic hemodialysis [148]

* K_d_ is the dissociation constant.

## 4. Extracellular Actin and Immune Processes

In addition to the acute toxic effects of excess F-actin concentrations in the bloodstream, there are effects of lower or local levels of this protein.

### 4.1. Participation in Antigen Cross-Presentation

Extracellular actin is perceived by nonspecific components of the innate immune system as a marker of cell damage and is involved in the initiation of the immune response [153,154]. Such molecules, which signal the immune system about damage, are classified as DAMPs—damage-associated molecular patterns or danger-associated molecular patterns [155,156]. Recognition of extracellular F-actin is carried out by DNGR-1 receptors (Dendritic Cell Natural Killer Lectin Group Receptor-1), which are also designated CLEC9A (C-type lectin domain family 9 member A) [153,157,158,159]. They belong to the C-type lectin receptor family [160], localized on the surface of dendritic cells (type 1 conventional dendritic cells—cDC1s) [46]. Dendritic cells, using F-actin, recognize and engulf cellular debris and use it for cross-presentation of viral and tumor antigens of dead cells to naive CD8(+) T cells [46]. In addition to DNGR-1, other receptors for various antigens are also involved in the cross-presentation process: Fc-receptors, CLEC7A (dectin 1), DC-SIGn (Dendritic Cell-Specific Intercellular adhesion molecule-3-Grabbing Non-integrin, CD209), LY75 (Lymphocyte antigen 75, DEC205, CD205) and mannose receptor 1 (CD206) [160]. In contrast to canonical presentation of an endogenous antigen, in the case of cross-presentation via major histocompatibility complex I (MHC-I), an exogenous antigen is presented on a dendritic cell [161]. The result of this process is cross-priming—the activation of naive CD8(+) T cells for clonal expansion and the formation of an effector phenotype [162]. Two mechanisms for preparing antigens for cross-presentation are considered: after capturing cellular debris, dendritic cells lyse it in phagosomes, where (1) they process the released antigens and load them with MHC-I peptides, or (2) antigens are transferred from phagosomes by unknown mechanisms into the cytosol and are cut into peptides in proteasomes, which are transported through TAP into the cisternae of the endoplasmic reticulum, where they are bound by MHC-I molecules [163,164]. It has been shown that the outcome of F-actin recognition by the DNGR-1 receptor depends on the alternative signaling cascade it activates. Upon activation of spleen tyrosine kinase (SYK), phagocytized debris is directed to non-degradable, non-acidic, non-lysosomal compartments, which is a key step in cross-presentation required for optimal generation of resident memory CD8(+) T cells. On the other hand, activation of H2 domain-containing phosphatase 1 (SHP-1) triggers a chain of events that limit tissue inflammation by suppressing the recruitment of neutrophils to inflammatory foci [46]. Interestingly, the F-actin-associated motor protein myosin II enhances the binding of F-actin to DNGR-1 [165], and plasma gelsolin competes with this receptor for binding to extracellular F-actin and weakens the processes of antigen cross-presentation [166]. The mechanisms of actin participation in the immune response are evolutionarily ancient. This is evidenced by the ability of arthropod fat body cells to recognize G- and F-actin and induce a response similar to the acute phase response in vertebrates [167]. Thus, extracellular actin serves as an important marker of dead cells for the immune system. It enables antigen-presenting cells to take up cellular fragments and, through mechanisms involving the cross-presentation of intracellular antigens, activate cytotoxic CD8(+) T cells. This process is a crucial link in the development of an immune response against viruses and intracellular bacteria.

Extracellular actin binds to MARCO (macrophage receptor with collagenous structure), which inhibits macrophage uptake of *S. pneumoniae*, *S. aureus*, and *E. coli* [168]. MARCO is a scavenger receptor that is responsible for the binding and phagocytosis of not only bacteria, but also cellular debris, tumor cells [169] and non-opsonized particles from the environment [168]. Thus, observed inhibition of MARCO will increase susceptibility to secondary infections and decrease mechanisms of anti-tumor immunity. On the other hand, blocking MARCO helps reduce the negative effects of chronic inflammation. This principle underlies the use of polyguanylic acid (PolyG—a MARCO inhibitor), which exerts an antifibrotic effect in experimental silicosis in rats [170]. The benefit or harm of this effect of extracellular actin will likely depend on its concentration and the accompanying conditions, which are determined by the specific features of concurrent pathological processes and the body’s resistance systems.

### 4.2. Interaction of Extracellular Actin with Soluble Components of the Immune System

The possibility of interaction of actin microfilaments with C1q has been established, which can activate the complement cascade due to non-antibody-mediated activation of C1q [171]. This process is the classical pathway of complement activation [172]. This process represents the classical pathway of complement activation. Its functions include the clearance of dead cells [173], opsonization and lysis of pathogens [174], and the amplification of vascular inflammation [175]. Excessive amplification of the inflammatory process in blood vessels, driven by complement activation, may be the underlying cause of the vascular lesions observed following the experimental intravenous administration of actin, as discussed earlier (see Section 2). Furthermore, activation of the complement system enhances the activity of tissue factor, which triggers the extrinsic pathway of blood coagulation [176]. This may serve as an additional mechanism underlying the thrombogenic effects of extracellular actin. Of interest is the ability of F-actin to bind the antibacterial peptide LL-37 from the cathelicidin family (cathelicidins) [177]. This peptide is a “natural antibiotic”—a means of antimicrobial protection and is produced mainly by neutrophils, myeloid cells of the bone marrow and epithelia [178,179]. Polymerized actin can also bind histones (protein components of nucleosomes) in the extracellular space, released from cells during their death. In the extracellular space, these proteins perform an antimicrobial function. Binding to F-actin protects them from bacterial proteases, as does the LL-37 peptide, but at the same time inhibits the antibacterial effect of these proteins [180]. Thus, extracellular actin, on the one hand, increases the stability of the antibacterial peptide LL-37, thereby enhancing its bactericidal properties and bolstering the body’s defense against bacteria. On the other hand, extracellular actin inhibits the antimicrobial action of histones, which reduces the level of antibacterial defense. Further research is required to determine whether this paradoxical effect of actin on bactericidal proteins holds any biological significance, or if it is merely a consequence of the incidental complementarity of these biomolecules.

### 4.3. Interaction of Extracellular Actin with Bacteria

Another role of extracellular actin is its binding to the surface proteins of some bacteria. This has been shown for a number of microorganisms pathogenic for humans and other mammals: phosphoglycerate kinase (PGK), *Streptococcus agalactiae* [181,182], P97 cilium adhesin, *Mycoplasma hyopneumoniae* [183], MSG1 (*Mycoplasma suis* glyceraldehyde-3-phosphate dehydrogenase-like protein 1), *Mycoplasma suis* [184]. The biological significance of this phenomenon is interesting from the point of view of the mechanisms of resistance and susceptibility to infections. The authors of these publications consider actin as a receptor used by bacteria for attachment to host cells.

In a study by SL Sandiford et al. (2015) [185], the involvement of extracellular G- and F-actin in immunity to Gram-negative bacteria in insects (*Anopheles gambiae*) was characterized in detail. It was shown that both forms of actin mediate phagocytosis of bacteria with equal efficiency by binding to their surface and interacting with the extracellular MD2-like immune factor AgMDL1. Direct antibacterial activity is predominantly possessed by G-actin, which is able to more easily integrate into the bacterial wall [185].

It has been found that extracellular actin stimulates an increase in the production of hemolysin by *Escherichia coli* (pathogenic and laboratory strains carrying hly genes), which may be important for the development of infections caused by pathogenic serotypes of *E. coli* [186]. It has been shown that F-actin and DNA from dead neutrophils, when included in the composition of *Pseudomonas aeruginosa* biofilms, can enhance their formation [187].

Furthermore, the interaction between the cytoskeleton and bacterial toxins—which can induce either the depolymerization or the stabilization of F-actin—is being actively studied. There are numerous reviews on this subject that examine the effects of various bacterial toxins on the actin microfilament system within target cells [188,189,190,191]. However, the effects of these toxins on extracellular actin have not been addressed, which presents an interesting avenue for future research.

In general, direct interactions between bacteria and extracellular actin are primarily viewed by considering F-actin as a biopolymer—one that bacteria perceive as both a signal and a receptor, which can facilitate the progression of an infectious process. The evidence regarding a direct bactericidal effect of extracellular actin in humans, as well as the potential for this effect to be modulated (e.g., through dissociation or stabilization) by bacterial toxins, requires further direct scientific validation.

### 4.4. Extracellular Actin and Extracellular Traps

The presence of actin has been shown in the so-called “extracellular traps” (Ets) [192]. These structures represent macromolecular networks of nuclear and mitochondrial DNA with a variable spectrum of proteins that perform bactericidal functions. The most typical of these are histones H2A, H2B, H3, and H4, neutrophil elastase, myeloperoxidase, proteinase 3, calprotectin, lactoferrin, azurocidin (AZU1), cathelicidin, and lysozyme C [193]. In humans and other mammals, the release of “extracellular traps” (ETosis) [194] is considered primarily as one of the tools of immune cell reactions: neutrophils—NETosis [192], eosinophils—EETosis [195], basophils—BETosis [196], mast cells—MCETosis [197], plasmacytoid dendritic cells—pETosis [198], microglial cells—MiETosis [199], monocytes/macrophages—METosis [200] and T-lymphocytes—TETosis [201]. Neutrophil extracellular traps (NETs) are the most studied in terms of the mechanism of production, composition and role in the pathogenesis of various diseases. A distinction is made between suicidal and vital NETosis [202,203]. Intracellular actin structures have been shown to play an active role in Ets production [204]. Data on the location and role of actin in extracellular traps are controversial. There are publications discussing the presence of actin in extracellular traps [192,205,206,207,208], and articles that do not mention it [193,209]. Some studies aimed at identifying actin in NETs note its absence in these structures [210,211,212]. This reflects the heterogeneity of the composition of Ets produced by different cells under different conditions. It should also be taken into account that actin can be in a complex with Ets, being released from dead cells, independently of the ETosis process. The specific role of this protein as a component of Ets has not yet been studied. Nevertheless, the inhibitory effect of actin on DNase I has been established [213], and the resistance of NETs enriched in actin microfilaments to destruction by nucleases has been shown [214]. Inhibition of DNase activity of blood serum by actin released from damaged tissues during burns and mechanical injuries has been established, leading to excessive and unregulated formation of NETs [215,216,217]. There are two aspects to increasing the stability of Ets: on the one hand, it promotes a more effective fight against pathogenic microorganisms using various strategies to escape from Ets [218], and on the other hand, Ets are integrated into the pathogenetic mechanisms of the disease and increase the likelihood of its complications [219]. To date, NETs in complex with actin have been identified in cystic fibrosis in the lumen of the respiratory tract [220], in the synovial cavity in experimental rheumatoid arthritis [221]. It has been shown that NETs are part of thrombi and the use of DNase I accelerates tPA-induced thrombolysis [222]. Data from animal experiments indicate that NETs are capable of directly inducing thrombosis [223]. In sepsis, excessive NETosis induces immunothrombosis, disseminated intravascular coagulation and multiple organ failure [215]. Despite the pronounced protective functions of NETs in capturing and neutralizing SARS-CoV-2 [224], the role of the excessive production of these structures in the development of COVID-19 complications is widely discussed: cytokine storm, macrophage activation syndrome, immunothrombosis, acute lung injury, and acute respiratory distress syndrome [202,225,226]. The involvement of NETs in the development of vaccine-mediated thrombotic thrombocytopenia (VITT) has also been shown [227]. Of interest are the interactions of NETs with the peptide LL-37, which, as we wrote above, is stabilized by actin from degradation. It has been shown that LL-37 promotes an increase in the level of NETs in blood plasma and peritoneal fluid. This peptide also protects NETs from DNAse degradation. A direct correlation between LL-37 and NET levels in plasma has been shown in COVID-19 patients, suggesting a potential role for LL-37 in the development of NET-associated immunothrombosis [228]. It has been established that, in addition to NET-derived material, DNA enters the systemic circulation through release from cells undergoing damage and stress [229,230,231]. This biopolymer also acts as a DAMP and can serve as an inducer of immunothrombosis [232]. DNA released from damaged cells has been shown to be closely associated with F-actin [56]. This association hinders its natural degradation by DNases, thereby enhancing its thrombogenic effect. Thus, extracellular actin is capable of stabilizing extracellular traps and prolonging their protective effect, which is important for effective antibacterial defense. An excess of actin can inhibit the clearance of extracellular traps by the body and lead to the negative effects associated with excessive NETosis.

### 4.5. Actin in Cytonemes

Discovered protective structures of leukocytes, as well as other cells, including non-immune cells [233], consisting of F-actin—cytonemes produced by mast cells. These cells, upon contact with pathogenic microorganisms, release actin filaments into the external environment, carrying antimicrobial agents (cathelicidins and neutrophil elastase), which, like neutrophil DNA traps, capture and destroy bacteria [234]. These structures (130–250 nm in diameter) are covered by the plasma membrane, but are very vulnerable: they are subject to shedding and decay [235,236,237], as a result of which they can serve as a source of extracellular F-actin.

Thus, extracellular actin has a fairly broad spectrum of interactions with the immune system and potentially pathogenic microorganisms. These interactions can enhance or weaken immune responses, stimulate or suppress infection, and can also cause more severe disease progression or the development of complications.

## 5. Interaction of Actin Clearance System Components with Inflammatory Mediators

The ability of components of the actin clearance system to interact with inflammatory mediators is another link in the mechanism of development of negative effects of extracellular actin due to the formation of its imbalance. It has been found that Gc-globulin binds arachidonic acid [130]; gelsolin binds lysophosphatidic acid, sphingosine-1-phosphate and phosphoinositides [43,112,238,239], platelet activating factor [240] and bacterial lipopolysaccharides [241], lipoteichoic acid [242]. Such interactions can be competitive: complex formation with actin reduces the ability of Gc-globulin to bind arachidonic acid [130], and the binding of sphingosine-1-phosphate and lipoteichoic acid by gelsolin reduces the actin-depolymerizing activity of gelsolin [112,242]. In general, the interactions of gelsolin and Gc-globulin with inflammatory mediators can be weakened by actin binding, which will disrupt their immunomodulatory effects [43]. Gc-globulin has been shown to enhance neutrophil chemotaxis in response to complement fragment C5a [243], and the Gc-globulin—G-actin complex is also an active cofactor of neutrophil chemotaxis [244].

Unlike the two classical components of the actin clearance system discussed previously, thymosin β4 is a protein with very pronounced multifaceted regulatory functions in the extracellular environment. In addition to binding G-actin, it has the ability to influence NF-κB signaling cascades, Toll-like, PI3K/Akt/eNOS, Notch and TGF-β. Depending on the regulated signaling pathway, thymosin β4 can exert proapoptotic, antiapoptotic, proliferative, immunomodulatory, antioxidant, angiogenic, regenerative and antifibrotic effects [245,246]. Any functions of profilin-1 in the extracellular environment not related to actin sequestration are currently unknown.

Thus, proteins responsible for actin clearance are also involved in the regulation of immune and regenerative processes, which should be taken into account when using them as predictive biomarkers and drugs.

## 6. Antibodies to Actin in the Development of Autoimmune Diseases

The role of actin as an autoantigen is discussed in relation to many autoimmune processes: hepatitis, celiac disease, idiopathic nephrotic syndrome, hemolytic anemia [247,248,249,250,251]. These diseases have individual profiles of their specific major autoantibodies [252,253]. Antibodies to actin may be either key in their case or of secondary importance and be associated with complications. In general, the formation of autoantibodies is a consequence of the activation of autoreactive lymphocyte clones that have avoided elimination by central tolerance processes. Mechanisms of peripheral tolerance implemented through inhibitory molecules, anergy, ignoring, and suppression by Treg cells are responsible for preventing their activation [254]. Weakening of these mechanisms in combination with processes of tissue damage and inflammation, infection by microorganisms (carrying antigens structurally similar to autoantigens), and factors of genetic predisposition leads to the development of an autoimmune process [255].

Autoimmune hepatitis type I is the most common disease associated with the production of antibodies to F-actin [256,257,258]. Moreover, in this case, anti-actin IgG serves as a diagnostic criterion for this form of hepatitis [259,260,261,262], including in childhood [263,264]. The association of anti-actin antibodies with HLA B8 and DR3 in patients with autoimmune hepatitis. In this disease, antibodies to actin indicate an unfavorable outcome [265] and are associated with low susceptibility to treatment with corticosteroids (16 vs. 4%), higher probability of hepatic death or requirement for liver transplantation (20 vs. 4%) [266]. High titers of antibodies to actin (>1:80, by IIF) are associated with the activity of the inflammation in liver tissue [267].

The presence of IgG to actin has been shown in children with obesity and non-alcoholic fatty liver disease (13.8%) and non-alcoholic steatohepatitis (15.5%) [268]. Anti-actin antibodies are recorded in the blood in toxic hepatitis induced by chlomethacin (63%) [269], and in the case report of consumption of Khat (*Catha edulis* (Vahl) Forssk. ex Endl.) [270]. Also, anti-F-actin antibodies have been detected in primary biliary cirrhosis (53%) and in alcoholic liver disease (19–25%) in the context of the development of an autoimmune process [271,272], and the presence of antibodies to actin correlated with high levels of IL-17A, IL-17F, IL-22 in blood and with alcoholic liver disease progression.

An autoimmune response to F-actin has been established in amyotrophic lateral sclerosis [273], and the level of IgG to F-actin is positively correlated with patient disability and with the duration and severity of this disease [274].

Actin has been shown to be involved as an autoantigen in cardiovascular diseases. A positive correlation has been shown between antibodies to actin and the thickness of the intima-media and adventitia of the carotid arteries in atherosclerosis [275], and between IgG to actin (45%) and myosin (42%) and the severity of damage to the cardiac muscle in acute coronary syndrome [276]. In postcardiac syndrome, antibodies to actin and myosin are formed after various injuries (aortic and mitral valve replacements, aorta-coronary bypass, acute myocardial infarction, acute viral myocarditis) to the cardiac muscle [277]. The surgical postcardiac syndrome is characterized by the presence of anti-actin IgG in 25% and anti-myosin IgG in 33% of patients. A positive correlation has been revealed between the intensity of post-cardiac injury syndrome and anti-actin IgG level in sera of patients with chronic ischaemic heart disease after coronary artery bypass grafting [278]. Cardiac surgery induced the most pronounced humoral immune response (IgM and IgG) to actin and myosin compared to acute myocardial infarction and viral myocarditis [279]. It has been shown that dilated cardiomyopathy induced by an autoimmune process [280] and by Emery-Dreifuss muscular dystrophy [281] is accompanied by an autoimmune response to troponin I, tropomyosin and actin. Antibodies to actin have been detected in the blood of newborns with Kawasaki syndrome (74%) [282].

IgA to actin (IgA-AAA) has been detected in celiac disease [283,284] and is a marker of disruption of epithelial architecture [285], severity of damage to the small intestinal mucosa [286] and villous atrophy [287,288,289]. The main diagnostic marker of celiac disease—anti-endomysial IgA (which predominantly includes IgA to transglutaminase) does not correlate with intestinal mucosal features [290]. The presence of IgA-AAA in the blood serum of patients depends on the severity of histological damage to the intestinal mucosa. Thus, IgA-AAA was detected by IIF in 25.7% of patients with celiac disease, but 77.8% of these IgA-AAA-positive patients had histological type 3c, and 22.2% had type 3 b [283]. The comparison of the ELISA assay with IIF revealed the same pattern of patient distribution, with different sensitivity in type 3a: 5.5% vs. 25% IgA-AAA-positive patients with type 3a, 27.5 vs. 34.4% with type 3 b, and 78.8 vs. 75% with type 3c detected with IIF and the ELISA assay, respectively [288]. It has been established that after a two-week gluten challenge, when the concentration of serum intestinal-fatty acid binding protein (I-FABP) increases, the level of IgA (including IgA to actin) increases on the 28th day, already upon returning the patient to a gluten-free diet [291]. The disappearance of IgA to F-actin after switching to a gluten-free diet indicates restoration of the intestinal mucosa [290]. The presence of IgA to actin has been shown in dermatitis herpetiformis, an extraintestinal manifestation of celiac disease [292], while IgG to microfilaments in the blood of patients may also be present, but without showing a clear relationship with dermatitis [293]. This lack of an association between anti-actin IgG and dermatitis—in contrast to the association seen with IgA—may be explained by the longer half-life of IgG (21 days) in the systemic circulation compared to that of IgA (4–7 days) [294]. Anti-actin IgA has also been recorded in the spectrum of autoantibodies in the blood of patients with IgA nephropathy [295]. IgA to actin has been detected in primary sclerosing cholangitis. IgM and IgA to actin were found in oligoarticular juvenile idiopathic arthritis [296].

In many autoimmune processes, entire groups of antibodies (primarily detected via indirect immunofluorescence) are formed, directed against antigens of certain cells, subcellular or molecular complexes (neutrophils, Ets, smooth muscle cells). It has been shown that autoantibodies to actin are part of the complex. ANCA (antineutrophil cytoplasmic antibody), characteristic of autoimmune neutropenia, systemic vasculitis [297]: Wegener’s granulomatosis (vasculitis of small and medium vessels with granuloma formation) and microscopic polyangiitis (vasculitis of small vessels without granuloma formation) [298], autoimmune hepatitis [256,257], primary sclerosing cholangitis [299], ANCA-associated glomerulonephritis [300]. The presence of antibodies to actin in ANETA (anti-NET antibody) complexes was detected in microscopic polyangiitis [301], ASMA (anti-smooth muscle cell antibodies) in microscopic polyangiitis [302] and autoimmune hepatitis type 1 [247,302,303], and ACPA (anti-citrullinated protein antibodies) in rheumatoid arthritis [304].

Antibodies to actin are also formed in infectious diseases. It has been shown that the level of IgG to F-actin in the blood of patients with hepatitis C is positively correlated with the intensity of the pathological process in the liver tissue (ELISA) [305] and associated with its more pronounced biochemical and histological activity (IIF) [306]. A high frequency of detection of antibodies to actin (ASMA, detected with IIF) in HCV/HIV-coinfected patients has also been shown [307]. These antibodies are also registered in HIV infection without HCV coinfection [305]. Cases of the development of autoimmune or polyautoimmune processes after COVID-19 infection, accompanied by the formation of antibodies to actin, have been identified. Lee et al. (2023) [308] describe a patient with seronegative autoimmune hepatitis that developed after COVID-19 infection. The process was characterized by elevated levels of antibodies to F-actin and the absence of antibodies to nuclear antigens, mitochondria, neutrophils, liver microsomes, kidneys, and soluble liver antigen [308]. AI Shorbagi et al. (2023) [309] report on a previously healthy vaccinated patient who was diagnosed with chronic colitis (resembling ulcerative colitis), autoimmune pancreatitis, and immune-mediated hepatitis 2 months after COVID-19 infection (Anti-smooth muscle antibody, actin) [309]. One of the main reasons for immunizing patients to F-actin during COVID-19 infection is the ability of antibodies to SARS-CoV-2 to react with various human tissue antigens, including F-actin [310,311].

The role of anti-actin antibodies in the pathogenesis of the aforementioned pathological processes is an intriguing question. Although this issue is generally considered to be insufficiently studied, it is believed that anti-actin antibodies do not exert a direct cytotoxic effect; rather, they are associated with processes of cytolysis, the intensity of which is reflected by their appearance or an increase in their levels [312,313]. In summary, extracellular F-actin is involved as an autoantigen in many autoimmune diseases. Immunoglobulins M, G, and A are formed against it. These antibodies can be used to develop predictive diagnostic technologies.

## 7. Discussion

The traditional role of actin as the main component of the actin cytoskeleton, an intracellular system that controls many functional aspects of cell structure and motility, is widely known [1]. However, this protein can also be present in the extracellular environment of the body, where it interacts with its various components. Actin is released from the cell during stress, damage, and death [47]. Thus, an increased level of actin in the systemic circulation always indicates intense cytolytic processes in the body and is recorded during ischemic, mechanical, thermal, and toxic tissue damage [57,58,59]. Due to its ability to interact with various cellular receptors, soluble and polymeric biomolecules, actin can induce a wide variety of physiological and pathological reactions in the extracellular environment of the body. Among the most detrimental effects of F-actin are thrombosis, immunothrombosis, excessive inflammation, obstruction and vascular injury [48,58,63,66]. This objectively characterizes the predictive potential of extracellular actin. The high risk of developing adverse complications caused by extracellular actin predetermines the importance of the system of its clearance from the extracellular environment of the body. This system includes components specialized for systemic circulation (plasma gelsolin, Gc-globulin) and universal for cells and the extracellular environment (thymosin 4β, profilin-1) [59,67,69,86,90]. The decrease in the levels of these proteins can lead to a disruption of actin clearance and the development of its negative effects. Multiregulatory functions, characteristic in addition to actin clearance for gelsolin, Gc-globulin and thymosin 4β, even more closely link an increase in F-actin levels and changes in the concentrations of these proteins with the induction and development of the inflammatory process [43,112,130,240,245]. Despite the large amount of information on the negative effects of extracellular actin, this protein also has a number of beneficial functions. Cross-presentation of intracellular antigens, mediation of phagocytosis, stabilization of extracellular traps, antibacterial peptides and thrombi are processes that, at low (physiological) local levels of actin production, are important protective mechanisms [46,177,185,214]. When the concentration of extracellular actin exceeds the physiological value, increased resistance to the timely utilization of extracellular traps and thrombi by the body will contribute to the development of excessive inflammation and immunothrombosis observed in severe infectious diseases (including COVID-19), sepsis and vaccine-mediated thrombotic thrombocytopenia [202,214,225,227]. In addition to its multifaceted effects on components of the immune system, actin can directly enhance the infectious process, since it serves as an adhesion receptor for some pathogenic bacteria, stimulates the production of bacterial toxins and promotes the growth of biofilms [182,183,186,187].

Another aspect of the effects of extracellular actin is a wide range of autoimmune processes in which extracellular F-actin plays the role of an autoantigen [247,248,249,251]. The main driving factors in this case are the disruption of the processes of central and peripheral immunological tolerance to a given antigen, in combination with cytolysis, infection by microorganisms with proteins that mimic actin [254,255,310]. In autoimmune diseases, antibodies to actin often do not have a leading function, but can be diagnostic criteria for these processes or biomarkers of their enhancement [265,267,274,286,287,290,292,306]. In general, extracellular actin exhibits a paradoxical array of effects—some beneficial, others harmful, and some even potentially lethal to the organism. A logical explanation for the coexistence of such diametrically opposed functions lies in the concentration of extracellular actin and the efficiency of its clearance. By applying Hans Selye’s theory of stress [314] —a fundamental concept in normal and pathological physiology that spans virtually all levels of biological organization—one can characterize how varying concentrations of extracellular actin interact with components of the immune and other systems (Figure 2). (1) Eustress (physiological stress): low, localized concentrations of extracellular actin are present, which the clearance system can easily eliminate. In this state, actin facilitates the cross-presentation of intracellular antigens, stimulates phagocytosis, enhances the efficacy of extracellular traps and antibacterial peptides, and, overall, promotes normal immune responses to cell damage and infection. (2) Distress: the level of extracellular actin exceeds physiological limits, overwhelming the clearance system (decompensation). Under these conditions, the detrimental effects of actin predominate: it impairs the aforementioned antibacterial defense mechanisms, exacerbates the negative consequences of NETosis (the formation of neutrophil extracellular traps), and can even be exploited by bacteria to facilitate the progression of an infection. These processes can contribute to the development of infectious and inflammatory diseases. Furthermore, such concentrations of actin may allow it to be recognized as an autoantigen by autoreactive immune cell clones (should any be present and activated within the body). (3) Shock represents a complete loss of control over the concentration of extracellular actin by the system responsible for its clearance; this results in uncontrolled actin polymerization and a rise in its concentration. A clear illustration of this process is provided by experiments in rats involving the intravenous administration of G-actin, which led to uncontrolled actin polymerization, vascular damage, and thrombosis (likely driven by both platelet activation and the activation of C1q). These mechanisms may be one of the causes of mortality in sepsis, severe trauma, and other conditions accompanied by intense cytolytic processes. Developing a more comprehensive picture of the role of extracellular actin and its clearance system in physiological and pathological immune mechanisms requires further detailed research. Currently, there are certain methodological challenges in developing precise techniques for analyzing this system. For example, standard clinical ELISA protocols cannot distinguish between G-actin, F-actin, and actin bound in scavenger complexes; such differentiation requires additional separation methods for these molecular complexes [315,316]. Furthermore, to date, no unified protocol with specific recommendations has been established for assessing changes in extracellular actin; instead, evaluations may focus on F-actin, the F-actin/G-actin ratio [317], or the ratio of actin to proteins involved in its clearance—such as the actin/gelsolin ratio [318] or the actin/Gc-globulin complex [59]. As we discussed in Section 6, the analysis of anti-actin antibody levels within the diagnostic technologies used for autoimmune diseases also requires a standardization of approaches—given the variety of methods employed—as well as further research to clearly define the role of different classes of these antibodies in various pathological processes.

The protective and multiregulatory role of the extracellular actin clearance system in the body predetermines the use of its components for therapeutic purposes. For this purpose, technologies are being developed using recombinant gelsolin [319], Gc-globulin [320] and thymosin β4 [321] to correct various critical conditions in patients. Information regarding the prospects for the use of profilin-1 in the scientific community has not yet been presented. Based on in vitro experiments, the prospects for using gelsolin to stimulate wound healing are examined [322]. A study in rats with experimental crush syndrome revealed no therapeutic effect from the addition of gelsolin to a standard therapeutic solution. Nevertheless, the authors suggest that it would be worthwhile to investigate various dosages of this protein to gain a more detailed understanding of its potential efficacy [323]. In Sprague-Dawley rats with a burn injury model, intravenous administration of recombinant human gelsolin prevented an increase in pulmonary microvascular permeability [324]. Experiments on mice with a sepsis model demonstrate that replenishing plasma gelsolin is effective in reducing mortality in endotoxemic mice (increasing survival by 88%) [61]. Experiments on rats indicate that exogenous gelsolin can reduce the incidence of sepsis [325]. In a mouse model of primary pneumococcal pneumonia, recombinant human pGSN facilitated more effective bacterial clearance, reduced acute inflammation, and improved survival [326]. Studying the model of prematurity and bronchopulmonary dysplasia in mice has shown a promising role for gelsolin (inhalation) in prevention of chronic lung disease [327]. The use of recombinant human pGSN in experimental mice with autoimmune encephalomyelitis has suggested that gelsolin could be a potential therapeutic target for multiple sclerosis [328]. The potential therapeutic utility of gelsolin in atherosclerosis [329] and in Alzheimer’s disease [330] is being discussed. The results of preclinical toxicological studies of human Gc-globulin purified from blood plasma (as a potential medicinal product) indicate that the safety profile of the plasma-derived Gc-globulin meets the requirements for products intended for human use [320]. It is assumed that Gc-globulin might be a novel target for therapy of severe acute pancreatitis [331]. It is hypothesized that Gc-globulin replacement therapy in patients with fulminant hepatic failure or severe trauma may improve their chances of survival [332]. It was shown that treatment of neural injury with thymosin β4 enhances angiogenesis, neurogenesis, neurite and axonal outgrowth, and oligodendrogenesis, and thereby significantly improves functional and behavioral outcomes [333]. Thymosin β4 has demonstrated cardioprotective effects in preclinical models of myocardial infarction and in a randomized controlled trial, highlighting the drug’s potential for translational application [334]. Studies in animals have shown promise for further clinical investigation and the development of thymosin β4 as a novel, safe, and effective treatment for dry eye syndrome [335]. Phase II clinical trials have shown that thymosin β4 accelerates the healing of pressure ulcers, trophic ulcers (associated with venous stasis), and wounds in cases of epidermolysis bullosa [336]. Thus, the use of components of the clearance system for the treatment of various pathological processes is a promising area, though it is still in the early stages of development. These are primarily hypotheses and assumptions, experiments on animals, and a few isolated clinical studies—none of which yet provide a basis for developing a clear strategy for utilizing components of the extracellular actin clearance system in the treatment of various diseases. This requires a systematic approach that would make it possible to determine the efficacy of employing specific components of the clearance system or combinations thereof in dependence on the pathological process and its severity. It is essential to establish clear indications for the use of such therapy and, most importantly, to define its limitations, risks, and potential contraindications. Unfortunately, in the absence of ongoing research, authorities can only draw preliminary conclusions or suggest potential approaches to handling patient data. A rational approach to data utilization requires the development of diagnostic technologies capable of objectively assessing the state of the system in question and its capacity to compensate for extracellular actin. Overall, this issue necessitates a comprehensive analysis of experimental, translational, and scientific research.

Thus, developing a comprehensive conceptual picture of the role of extracellular actin in the implementation and regulation of the body’s immune responses is important for understanding the pathogenesis of many diseases characterized by tissue damage and the development of inflammatory and infectious processes. The development of diagnostic technologies that allow for an objective assessment of the system comprising extracellular actin and its clearance components, as well as clinical guidelines based on these technologies, will enable adequate prevention of hyperactinemia complications. Correcting the functions of the actin clearance system also holds promise for new medical developments. Studying the role and prevalence of actin antibodies is important for improving the diagnosis of the severity of autoimmune processes. All this will contribute to increasing the effectiveness of treatments for diseases associated with actin release from cells.

## Figures and Tables

**Figure 1:**
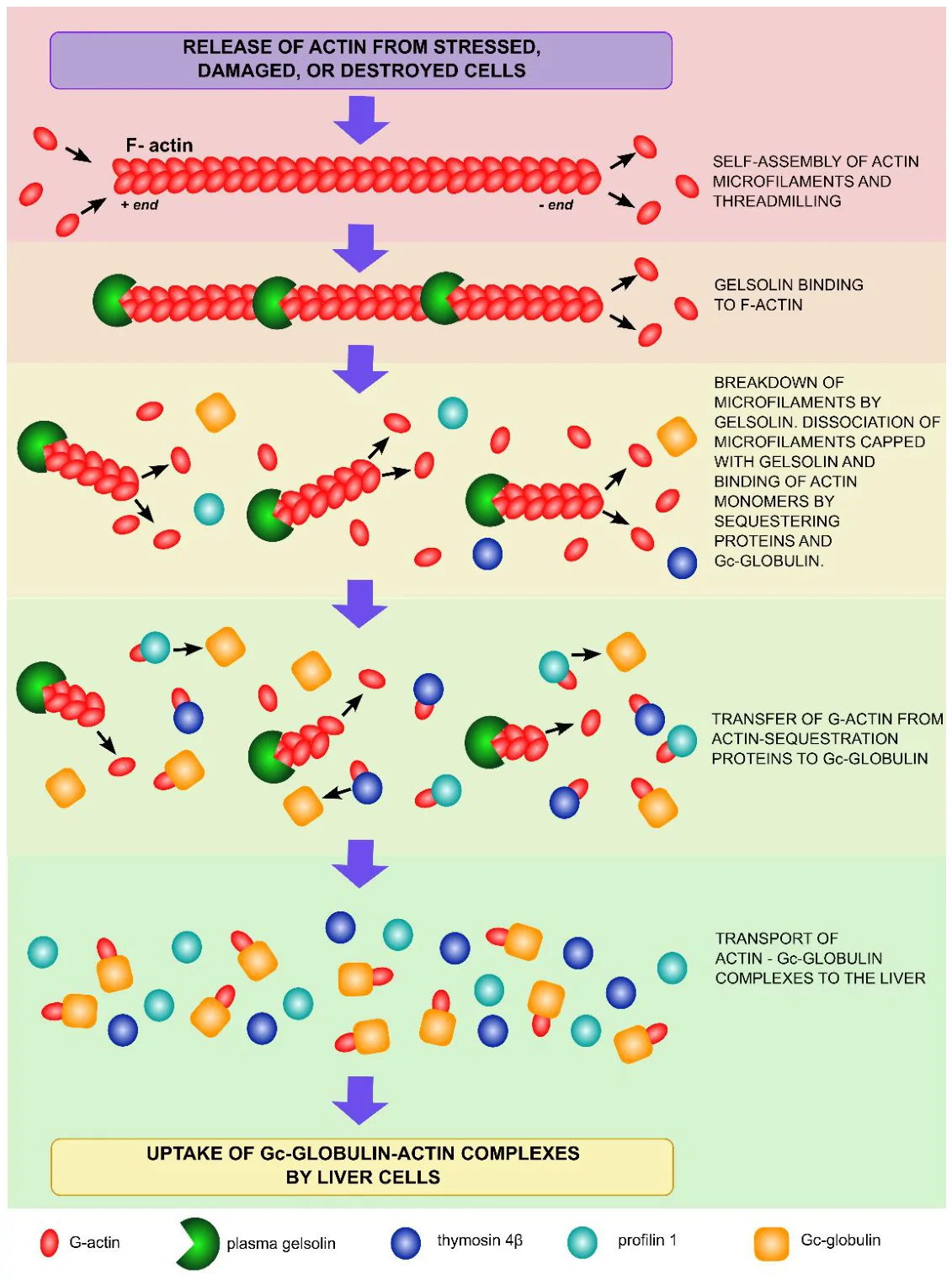
Extracellular actin clearance system. Actin, released from cells as a result of stress, damage, or death, is present predominantly in a polymerized form due to the self-assembly of microfilaments. At the plus end of the microfilament, actin monomers predominantly attach, while at the minus end, they dissociate. This process is called treadmilling. Plasma gelsolin, when attached to a microfilament, breaks it at the point of contact and remains bound to the plus end of the detached fragment (capping), blocking the addition of new monomers. This capped F-actin dissociates into monomers, which, upon dissociation, are bound by actin-sequestering proteins (thymosin β4, profilin-1) and Gc-globulin, which transports the bound monomers to liver cells for their uptake and removal from systemic circulation. Since Gc-globulin has a greater affinity for actin than thymosin β4 and profilin-1, the actin monomers captured by them will be transferred to Gc-globulin for subsequent transport to the liver.

**Figure 2:**
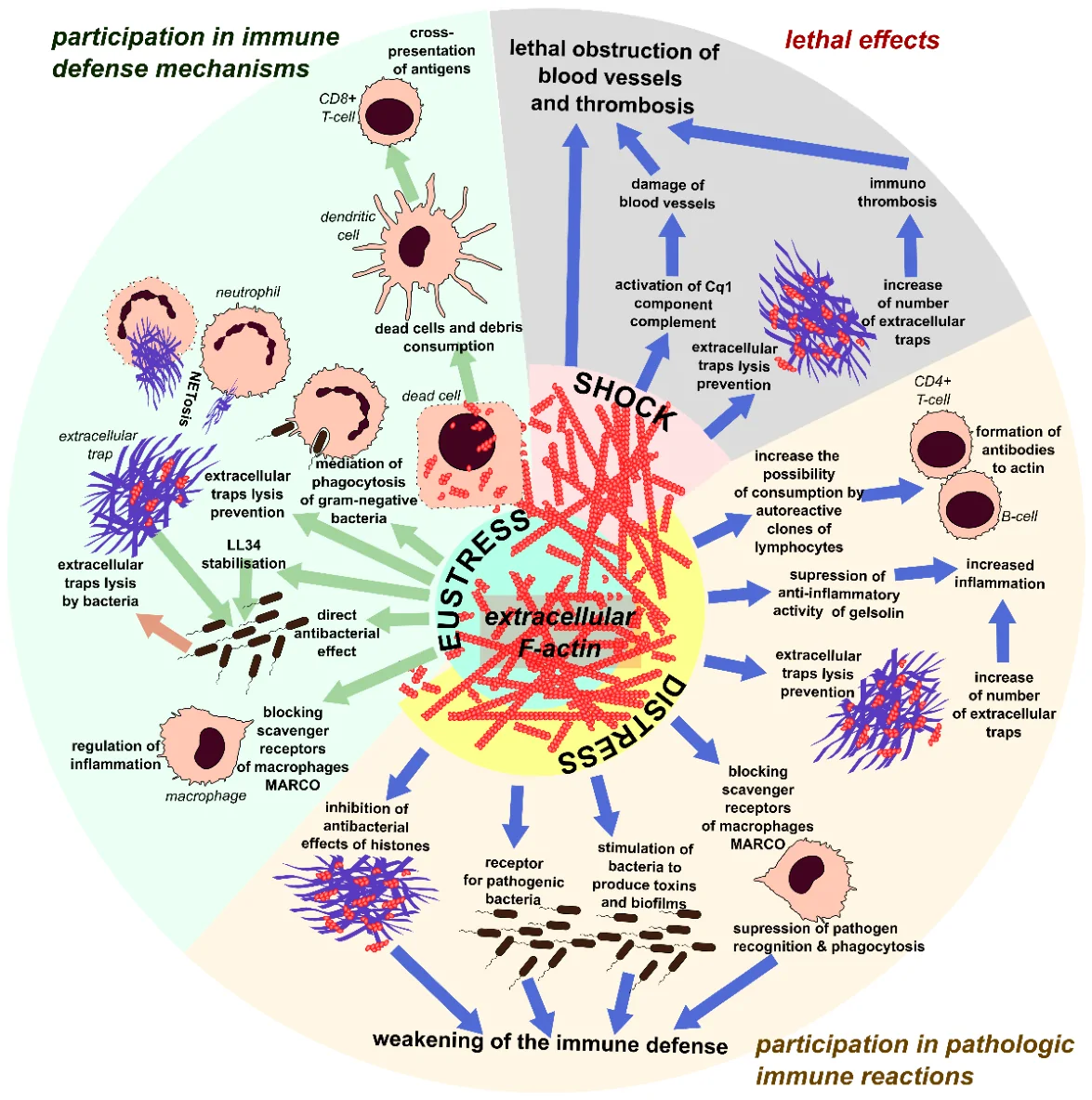
Multifaceted effect of extracellular actin on the immune system, hemostasis and the infectious process. Extracellular actin can exert a variety of effects in the body. On the one hand, at physiological concentrations effectively compensated by the clearance system (eustress), this protein can enhance immune defense against microorganisms and viruses by participating in the cross-presentation of intracellular antigens and stabilizing antibacterial peptides and extracellular traps. Its involvement in mediating the phagocytosis of Gram-negative bacteria and its direct antibacterial effect is debatable for humans and mammals, as this activity has only been studied in invertebrates. On the other hand, at higher, uncompensated concentrations (distress), extracellular actin can disrupt immune functions in the body: it can block MARCO scavenger receptors on macrophages, inhibit the antibacterial effect of extracellular histones, suppress the anti-inflammatory action of gelsolin, and, by blocking the timely disposal of extracellular traps, stimulate excessive inflammation and immunothrombosis. Actin can also directly stimulate infection, acting as an adhesion receptor for some bacteria and stimulating the production of bacterial toxins and biofilm growth. Actin has also been shown to act as an autoantigen in the development of various autoimmune processes. The most catastrophic effects on the body are those of high actin concentrations with complete loss of control over its regulation (shock): thrombosis, immunothrombosis, and vascular damage, which can lead to death.

## Data Availability

Data are contained within the article.

## Supplementary Materials

The following supporting information can be downloaded at: https://www.mdpi.com/article/10.3390/ijms272110049/s1.

## Abbreviations

The following abbreviations are used in this manuscript:

ACPA	anti-citrullinated protein antibodies
AgMDL1	MD2-like receptor and extracellular immune factor
Akt	Protein kinase B
ANCA	antineutrophil cytoplasmic antibody
ANETA	anti-NET antibody
ASMA	anti-smooth muscle cell antibodies
AZU1	Azurocidin
cDC1s	type 1 conventional dendritic cells—cDC1s
CLEC7A	C-type lectin domain family 7 member A
CLEC9A	C-type lectin domain family 9 member A
COVID-19	Coronavirus disease 2019
DAMPs	damage-associated molecular patterns or danger-associated molecular patterns
DC-SIGn	Dendritic Cell-Specific Intercellular adhesion molecule-3-Grabbing Non-integrin
DNase I	Deoxyribonuclease I
DNGR-1	Dendritic Cell Natural Killer Lectin Group Receptor-1
eNOS	Endothelial nitric oxide synthase
Ets	extracellular traps
Gc-globulin	group-specific component-globulin
HCV	Hepatitis C virus
HIV-1	Human immunodeficiency virus-1
I-FABP	intestinal-fatty acid binding protein
LL-37	37-amino-acid cationic antimicrobial peptide
LY75	Lymphocyte antigen 75
MARCO	Macrophage receptor with collagenous structure
MHC-I	major histocompatibility complex I
MSG1	*Mycoplasma suis* glyceraldehyde-3-phosphate dehydrogenase-like protein 1
NETs	Neutrophil extracellular traps
NF-κB	Nuclear factor kappa-light-chain-enhancer of activated B cells
NHYA	New York Heart Association (NYHA) Functional Classification
PGK	phosphoglycerate kinase
pGSN	plasma-type gelsolin
PI3K	Phosphoinositide 3-kinase
SARS-CoV-2	Severe acute respiratory syndrome coronavirus 2
SHP-1	H2 domain-containing phosphatase 1
SYK	spleen tyrosine kinase
TAP	Transporter associated with Antigen Processing
TGF-β	Transforming growth factor beta
VITT	vaccine-mediated thrombotic thrombocytopenia
